# Population dynamics of threatened Lahontan cutthroat trout in Summit Lake, Nevada

**DOI:** 10.1038/s41598-020-65992-0

**Published:** 2020-06-08

**Authors:** James B. Simmons, Teresa Campbell, Christopher L. Jerde, Sudeep Chandra, William Cowan, Zeb Hogan, Jessica Saenz, Kevin Shoemaker

**Affiliations:** 1 University of Nevada, Global Water Center, Department of Biology, Reno, Nevada United States of America; 2 Summit Lake Paiute Tribe, Sparks, Nevada United States of America; 3 University of Nevada, Department of Natural Resources and Environmental Science, Reno, Nevada United States of America; 4 University of California, Marine Science Institute, Santa Barbara, California, United States of America; 5 The Great Basin Institute, Reno, Nevada United States of America

**Keywords:** Conservation biology, Population dynamics, Ecology

## Abstract

Summit Lake, Nevada (USA) is the last high-desert terminal lake to have a native self-sustaining population of threatened Lahontan cutthroat trout (*Oncorhynchus clarkii henshawi*). From spring 2015 to fall 2017, we quantified adult abundance and survival and the total annual spawning run. Abundance and survival were estimated with mark-recapture using PIT tags, and the annual spawning run was estimated with PIT tag detections and counts of spawners. Adult abundance fluctuated from 830 (95% CI 559–1248) to 1085 (95% CI 747–1614), with no overall temporal trend, as a decrease in male abundance was generally offset by an equal increase in female abundance. Estimated mean adult survival was 0.51 (95% CI 0.44–0.58). The spawning run increased from 645 (2015) to 868 (2016), but then decreased slightly to 824 (2017, mean = 789 ± 118). Female spawners increased in 2016 but decreased slightly in 2017, whereas male spawners decreased each year. In addition, the proportion of adults that spawned each year increased overall. Our study suggests that the adult population remained stable although most of the study period included the recent, severe regional drought in the western United States (2012–2016).

## Introduction

Amid the backdrop of global biodiversity decline, North American freshwater fauna is declining five times faster than terrestrial fauna, including current extinction rates of freshwater fish 877 times greater than background rates^1–3^. Since the mid-1800s, habitat loss, overfishing and invasive species have severely altered western United States (US) freshwater fish communities^4^. Today climate change predictions for the large expanse of mountain ranges in the western US (increased climatic variability that will increase drought frequency, duration, and severity, and shift precipitation to more rain and less snow) threaten to compound the above disturbances^5–8^. These legacy, current and future disturbances combine into a formidable challenge for conserving western US freshwater fish biodiversity, often necessitating active management of fisheries that are susceptible to further decline and localized extinctions^9^.

Cutthroat trout (*Oncorhynchus clarkii* spp.) are salmonids native to the coastal and inland waters of western North America^10^. Consisting originally of approximately 14 subspecies, the historic distribution of cutthroat species ranged from Alaska to southern Texas and the Pacific coast to the Rocky Mountains^10,11^. Distribution and abundance of many subspecies have declined over the past century. Two subspecies are extinct and three subspecies are on the US endangered species list^10,12^. Cutthroat trout population dynamics research has been concentrated in the rivers and streams of the Rocky Mountains, the eastern side of the Intermountain Region (area between the Sierra Nevada/Cascade Mountains and the Rocky Mountains), and the Sierra Nevada Mountains^13–24^. Little is known about the population dynamics of cutthroat trout in lakes across the western US, especially the desert terminal mountain lakes of the Great Basin.

Lahontan cutthroat trout (*Oncorhynchus clarkii henshawi*, Lahontan cutthroat) was the top fish predator in ancient Lake Lahontan, the large inland sea during the Pleistocene that covered much of northwestern Nevada and small portions of northeastern California and southeastern Oregon. As Lake Lahontan desiccated over subsequent millennia, Lahontan cutthroat were restricted to remnant streams, rivers and lakes. By the mid-1800s, Lahontan cutthroat occupied 11 lakes, which ranged from the northwestern corner of Nevada to the middle portion of the Sierra Nevada Mountains in California, with six lakes in the Sierra Nevada Mountains and five lakes eastward in the high desert sagebrush steppe of the western Great Basin. Over the next one hundred years, Lahontan cutthroat were extirpated from 9 lakes. The mountain ecosystems of Independence Lake (California) and Summit Lake (Nevada), representing only 0.4% of the historic lake habitat, contain the last native self-sustaining adfluvial populations^25^. The precipitous decline of the adfluvial populations was a major reason the US Fish and Wildlife Service added Lahontan cutthroat to the US endangered species list in 1970^26^.

**Figure 1:**
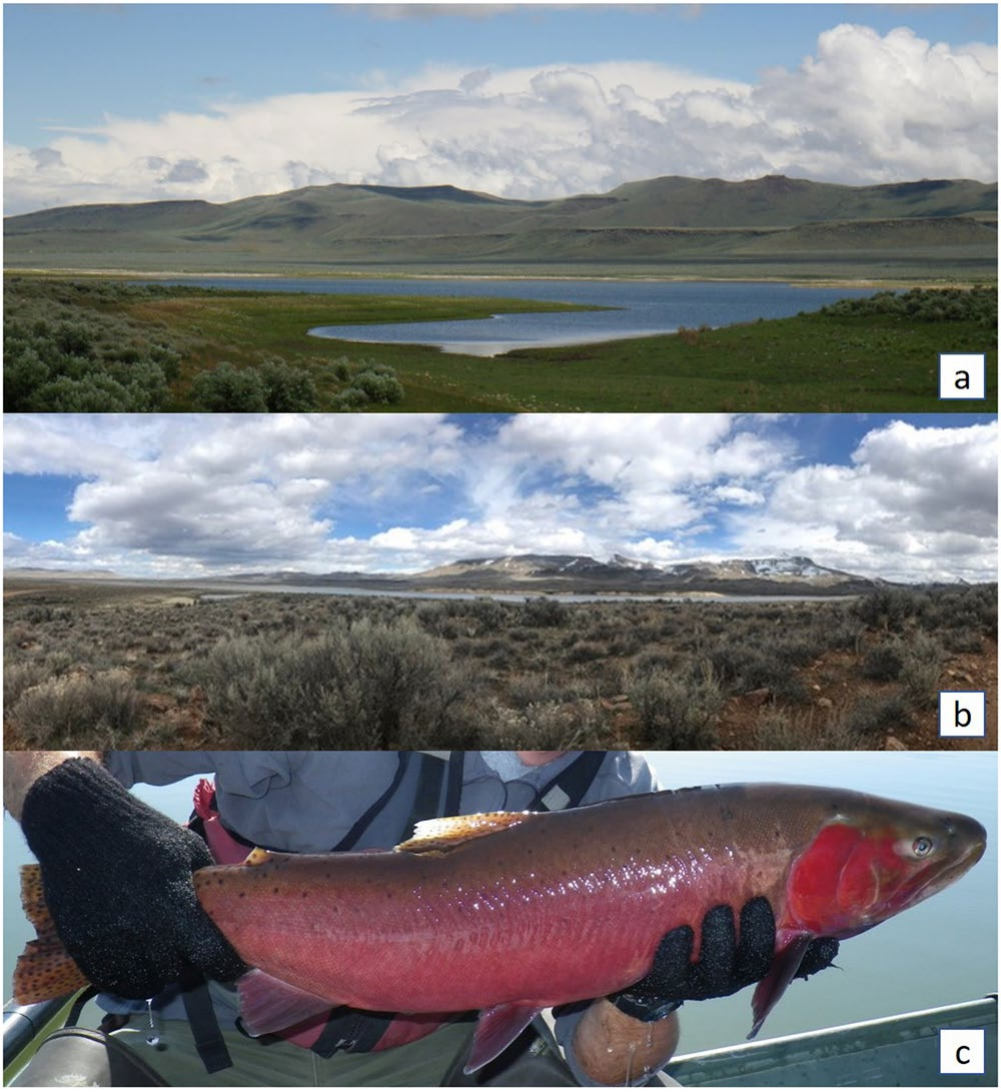
(**a**) Southwestern side of Summit Lake looking northeast; Summit Lake Mountain and watershed in the distance, (**b**) Northwestern side of Summit Lake looking southeast; high-desert sagebrush (*Artemisia tridentata*) steppe ecosystem - photo courtesy of Elizabeth Sisson, (**c**) Lahontan cutthroat caught during the lake mark-recapture effort of this study.

Summit Lake (Fig. 1a,b) is a desert terminal mountain lake in the Black Rock Range of the Black Rock Desert in remote northwestern Nevada. Approximately 8,000 years ago, a landslide blocked an area north of Pleistocene Lake Lahontan that had southward drainage, creating Summit Lake and potentially isolating its Lahontan cutthroat population^25,27^. The Lahontan cutthroat in Summit Lake (Fig. 1c) have been recognized as a unique subpopulation with significant cultural heritage for the region’s indigenous people^10,25,26,28,29^. The inextricable cultural connection between the Summit Lake Paiute Tribe (SLPT) and the fishery is reflected in the tribe’s original name, Agai Panina Ticutta, which translates as the Summit Lake Fish Eaters. Summit Lake (up to the high-water mark) is entirely contained within the Summit Lake Paiute Reservation, established in 1913^29^. The lake’s proximity to the Black Rock Desert, the population’s adaptation to the lake’s warm eutrophic alkaline waters, and the population’s genetic divergence from other Lahontan cutthroat populations warranted the population’s inclusion in the Northwestern Lahontan basin distinct population segment in the Lahontan cutthroat recovery plan^25,26,30–34^. In addition, the lake’s remote location and the restricted access to the watershed by the SLPT has kept the ecosystem and the Lahontan cutthroat population relatively undisturbed, buffering it from major disturbances (e.g., invasive salmonids, watershed development, public or commercial harvest) that have caused the decline of other Lahontan cutthroat populations^35^.

In this study, we addressed the following conservation questions about the population dynamics of the lake population: (1) what are the total, female and male adult abundances?, (2) what are the overall, female, and male adult survival rates?, and (3) what is the total number of spawners in the primary spawning tributary and what is their proportion of the adult population? We estimated abundance and survival with a robust design mark-recapture approach using passive integrated transponder (PIT) tags. The number of spawners was estimated by the detection of tagged individuals by a PIT antenna and the capture of tagged and non-tagged individuals in the spawning tributary during the spawning run.

## Results

The lake mark-recapture effort resulted in 1501 captures, with total captures per primary period ranging from 81 (post-spawn 2016) to 279 (fall 2015) (mean = 188 ± 70). We tagged 1168 individuals and 274 were recaptured (Table 1; Supplementary Datasets S1, S2).

**Table 1:** Summary counts of adfluvial Lahontan cutthroat captured during the lake mark-recapture effort at Summit Lake, Nevada, USA, 2015–2017.

Year	Primary Period	Number of secondary periods	New captures	Recaptures	Individual recaptures	Total captures
2015	Pre-spawn	8	212	47	33	259
	Fall	6	237	42	37	279
2016	Pre-spawn	2	171	68	37	239
	Post-spawn	4	48	33	30	81
	Fall	4	119	33	33	152
2017	Pre-spawn	2	153	52	52	205
	Post-spawn	4	87	30	25	117
	Fall	5	141	28	27	169
Total	—	35	1168	333	274	1501
Mean	—	4	146	42	34	188
SD	—	2	62	14	8	70

The New captures category is the number of new (without a Passive Integrated Transponder - PIT tag) individuals captured. The Recaptures category is the number of captures of PIT tagged individuals, including each capture of individuals recaptured more than once. The Individual recaptures category is the number of tagged individuals recaptured, not including the additional recaptures of individuals recaptured more than once. SD = standard deviation.

**Figure 2:**
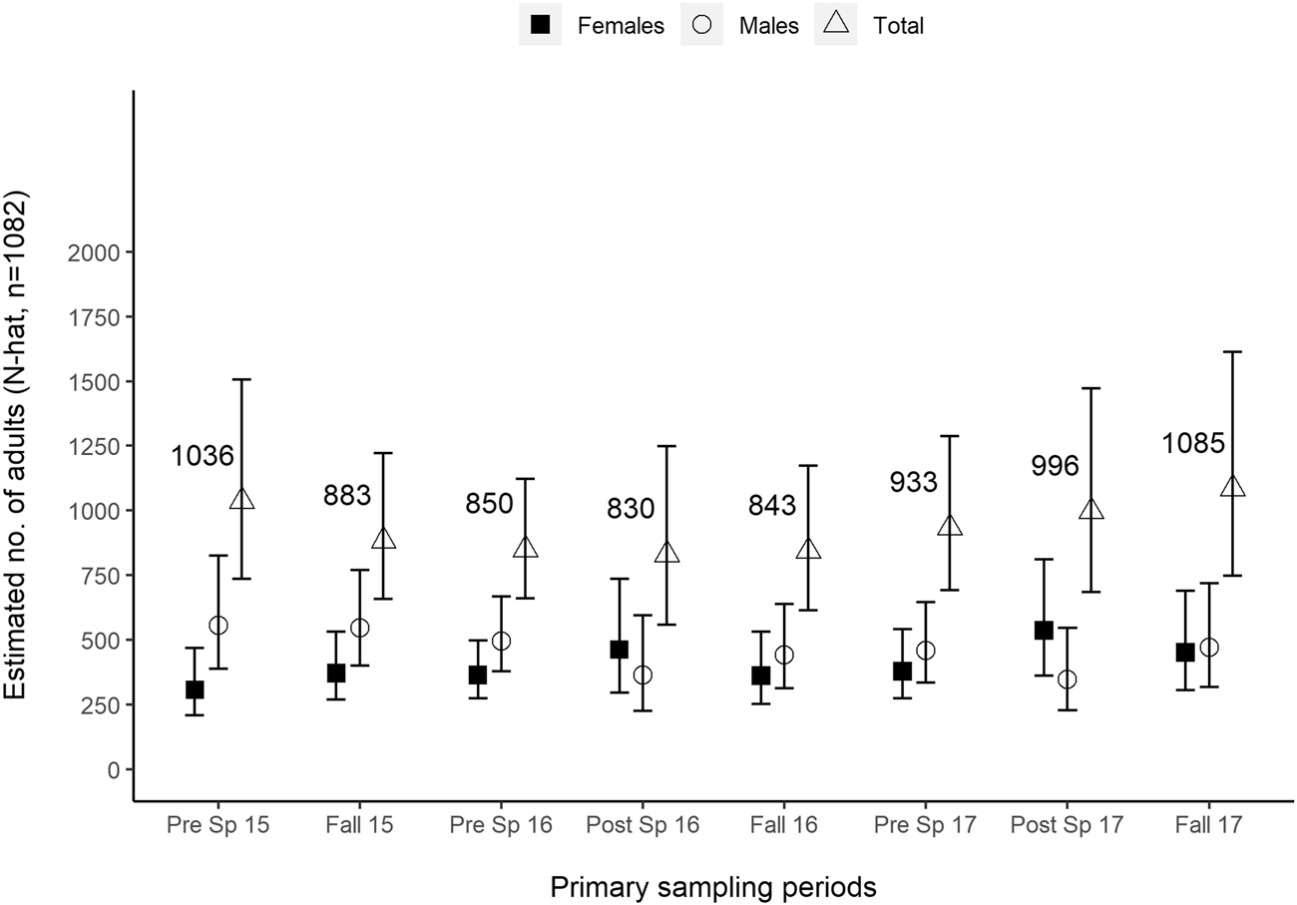
Adult abundance estimates derived from the adult (male, female, and unknown sex, ≥300 mm, n = 1082) adfluvial Lahontan cutthroat captured during the lake mark-recapture effort at Summit Lake, Nevada, USA, 2015–2017. The abundance ($\hat{N}$) estimates and primary sampling periods are located on the x and y axes, respectively. The abundance, female, and male estimates were derived from the top model of AIC (Akaike Information Criterion) model selection performed with the adult lake mark-recapture data (Table 2, Supplementary Tables S1–3). The error bars represent 95% confidence intervals.

The top model (Δ*AIC*_*c*_ = 0) for all adults (n = 1082, male, female, unknown sex) had time-invariant survival and lambda, and time-varying capture (*p*) and recapture (*c*) rates. In addition, lambda and recapture were modeled as a function of sex; and survival, lambda and recapture were modeled as a function of fork length (Table 2). From pre-spawn 2015 to fall 2017, estimated abundance was 1036 (95% CI 735–1507), 883 (95% CI 658–1223), 850 (95% CI 661–1122), 830 (95% CI 559–1248), 843 (95% CI 615–1173), 933 (95% CI 691–1288), 996 (95% CI 685–1473), and 1085 (95% CI 747–1614) (Fig. 2, Supplementary Table S1). Female abundance was estimated at 307 (95% CI 209–469), 371 (95% CI 269–531), 364 (95% CI 275–498), 462 (95% CI 295–735), 362 (95% CI 252–532), 379 (95% CI 274–541), 536 (95% CI 361–812), and 452 (95% CI 305–691) (Fig. 2, Supplementary Table S2). Male abundance was estimated at 556 (95% CI 388–826), 546 (95% CI 401–770), 495 (95% CI 378–667), 364 (95% CI 226–596), 442 (95% CI 312–639), 458 (95% CI 334–647), 348 (95% CI 227–547), 472 (95% CI 319–719) (Fig. 2, Supplementary Table S3). Annual survival (*S*_*A*_) was 0.51 (95% CI 0.44–0.58). The sex coefficients (logit) for lambda and recapture were −0.04 (95% CI −0.08 – −0.005) and 0.55 (95% CI 0.05–1.05), respectively. The fork length coefficients (logit) for survival, lambda, and recapture were −0.004 (95% CI −0.006 – −0.002), −0.002 (95% CI −0.002 – −0.002), and 0.006 (95% CI 0.003–0.009), respectively.

**Table 2:** Model selection results for seasonal survival (*S*_*s*_), lambda (λ), and capture (*p*)/recapture (*c*) rates of adult (male, female, and unknown sex, ≥ 300 mm, n = 1082) adfluvial Lahontan cutthroat captured during the lake mark-recapture effort at Summit Lake, Nevada, USA, 2015–2017.

*Model*	*AICc*^*a*^	*ΔAICc*^*b*^	*AIC weight*	*Model likelihood*	*K*^*c*^	*Deviance*
*S*_*s*_(.^*d*^*,fl*^*e*^)*-λ*(*.,sex,fl*)*,p*(*t*^*f*^)*,c*(*t,sex,fl*)	9550.44	0.00	0.71	1.00	25	9499.45
*S*_*s*_(*.,fl*)*-λ*(*.,sex,fl*)*,p*(*t,fl*)*,c*(*t,sex,fl*)	9552.22	1.78	0.29	0.41	26	9499.16
*S*_*s*_*-λ*(*a*^*g*^*,fl*)*,p-c*(*t,fl*)	9570.87	20.44	0.00	0.00	26	9517.81
*S*_*s*_*-λ*(*.,fl*)*,p-c*(*t,fl*)	9618.56	68.12	0.00	0.00	22	9573.79
*S*_*s*_(*a*)*,λ*(*a,sex*)*,p*(*t*)*,c*(*t,sex*)	9742.46	192.02	0.00	0.00	26	9689.39
*S*_*s*_*-λ*(*.,sex*)*,p-c*(*t,sex*)	9779.37	228.94	0.00	0.00	25	9728.39
*S*_*s*_*-λ*(*a*)*,p-c*(*t*)	9795.72	245.29	0.00	0.00	22	9750.96
*S*_*s*_(.)*, p-c*(*t*)	9856.58	306.14	0.00	0.00	18	9820.06
*S*_*s*_*-p-c*(.)	10290.48	4740.05	0.00	0.00	4	10282.45

^a^AIC (Akaike Information Criterion) for small sample size.

^b^Difference between model AIC_c_ and model with the lowest AIC_c_.

^c^No. of model parameters.

^d^(.) = rate constant across primary sampling periods.

^e^*fl* = fork length.

^f^*t* = parameter varies across primary sampling periods.

^g^*a* = parameter varies annually.

AIC (Akaike Information Criterion) model selection was performed. Sex and fork length (*fl*) are covariates.

From 2015–2017, respectively, the PIT antenna detected 238, 337, 344 spawning trout (mean = 306 ± 59), of which 77, 133, 159 were female (mean = 123 ± 41); 129, 191, 162 were males (mean = 160 ± 31); and 32, 13, 23 were unknown sex (mean = 22 ± 9). Concurrently at the fish weir, 268, 465, 433 spawning trout were caught (mean = 388 ± 105), of which 92, 171, 190 were female (mean = 150 ± 51); 169, 294, 239 were male (mean = 234 ± 63); and 7, 0, 4 unknown sex (mean = 3 ± 3). The proportion of tagged individuals was 0.38, 0.40, and 0.43 (mean = 0.40 ± 0.03). Thus, from 2015–2017 we estimated total spawning trout as 645, 868, 824 (mean = 789 ± 124), respectively, with total female spawners of 206, 338, 379 (mean = 307 ± 90), male spawners of 348, 494, 387 (mean = 410 ± 76), and unknown sex spawners of 91, 36, 58 (mean = 61 ± 27). The estimated proportion of adults that participated in the spawning run was 0.62, 1.0, and 0.88 (mean = 0.73 ± 0.23; with values above 1.0 restricted to 1.0).

## Discussion

Overall, the Summit Lake population was stable during our two year and 9-month study period. Population growth rate (lambda) differed by sex, with females exhibiting a slightly higher growth rate than males. However, this sex-specific difference in abundance trend did not appear to result in a severe sex imbalance. In addition, fork length had a negative effect on the population growth rate.

The apparent reduction in total adult abundance we observed during the first two-thirds of our study may have resulted from low snowpacks prior to and during this portion of our study period. From 2012 to 2014, Summit Lake snowpacks (measured in snow water equivalent or  SWE, the equivalent depth of liquid water) decreased by approximately 40% (Supplementary Table S4)^36^. Cutthroat fry recruitment has been shown to be positively correlated to the lower third approximately (negatively correlated for the upper two - thirds) of the range of snow - water content (via streamflow) in a spawning tributary’s watershed^37^. Summit Lake fry in 2012 would have started becoming adults in 2015. Consequently, the apparent decline in abundance during this period may be partially attributed to decreased fry recruitment from 2012 to 2014. However, the reason(s) is unclear for the seemingly opposite abundance trends for males and females. With no difference in male and female survival, differential recruitment between the sexes was probably the main driver, with skewed embryo survival a possible mechanism. Morán *et al*. found that brown trout (*Salmo trutta*) families with high embryo mortality tended to produce female-biased clutches of alevin^38^. With lower streamflow and higher water temperatures likely to have increased embryo mortality in Mahogany Creek from 2012–2016, adult recruitment may have been skewed toward females via this mechanism of skewed embryo survival^39,40^.

Sex did not seem to have a significant effect on total, female, and male survival. Cutthroat trout survival in this mountain lake is low compared to adult (total) or adult female survival from three other Lahontan cutthroat studies^14,41,42^. The Truckee River (Nevada) study had the lowest survival (0.36). The estimate was probably biased low by apparent survival (which is always lower than true survival), hatchery Lahontan cutthroat (which can exhibit lower fitness than wild conspecifics), small size (large juveniles or first year adults were used, which could have been subject to high competition and predation pressure from the more abundant wild populations of brown trout (*Salmo trutta*) and rainbow trout (*Oncorhynchus mykiss*)), or drought^25,41,43–46^. The Walker Lake (another mountain desert terminal lake in the region, Nevada) study estimated adult survival at 0.44 in 1999 (highest rate of their study)^42^. True survival was estimated, but hatchery Lahontan cutthroat were used; and at approximately 11 g/L, the total dissolved solid level in Walker Lake significantly decreased Lahontan cutthroat survival^47,48^. In contrast, the natural cutthroat trout population of mountain Independence Lake had the highest survival rate (0.68), but survival was estimated for females only^14^. Though wild invasive salmonids (primarily kokanee salmon, *Oncorhynchus nerka*) inhabit Independence Lake, the Lahontan cutthroat population is wild and had favorable snowpack during the study^49^. Accordingly, given the study designs and stressors that lowered survival in above Lahontan cutthroat studies, it is plausible that our estimate can be considered low and driven by a stressor. In addition, Summit Lake survival is in the lower half of a survey of annual survival rates from other trout studies^14,15,19,50–52^.

The female and male spawning numbers and proportions followed their respective abundance trends, but the total spawning numbers and proportions generally increased while total abundance first declined then increased to remain relatively stable for the study period. Like with abundance, snowpack size and timing and the potentially skewed recruitment (increase in females) may have driven the total spawning run numbers and proportions. Spawning runs have been shown to be positively correlated to the lower third approximately (negatively correlated for the upper two-thirds thereafter) of the range of spring water flow (via snow-water content) in the spawning tributary^37,53^. The Summit Lake spawning runs and proportions increased in 2016 and 2017 with consecutively larger snowpacks, and the spawning runs generally matched the snowpack trend from 2012 to 2017 (Supplementary Table S4)^36^. Moreover, the March - April snowpacks of 2015, which were the smallest March - April snowpacks during 2012–2017, probably contributed to the historically low spawning run at the fish weir in 2015. The small decline (from 2016) of the spawning run in 2017 may have been partially due to high SWE and water flows that year^53^. Finally, the spawning proportion for 2016 (1.00) is not realistic but can be viewed in the context of the confidence interval of the abundance estimate for the period, suggesting the proportion was higher than 2015 but like 2017.

Drought (along with invasive salmonids and agricultural dewatering) has been implicated in the decline of various species of river - or stream - dwelling cutthroat trout populations, but not adfluvial cutthroat populations^13–15,20,21,24^. However, taken together, our observations at Summit Lake from 2015–2017 (e.g., potential decline in adult abundance from 2015 to 2016, opposite female and male abundance trends, low annual survival, larger proportion of spawners the last two years, smallest spawning run on record at the fish weir) were likely driven by the severe sustained regional drought from 2012–2016^54^. Considering also the potential negative legacy effects of drought, what is the prognosis for the Summit Lake adfluvial Lahontan cutthroat population^55–57^? Populations naturally fluctuate with changing abiotic or biotic conditions or drift processes^43^. Thus, declining abundance during normal drought cycles and conditions may not be a concern. Other trout populations have comeback from precipitous declines and drought^58^. For the Summit Lake Lahontan cutthroat population in the lake, the increase of 2016 and 2017 spawning runs may signal a comeback, and in the short run, continued average to above average snowpacks and low anthropogenic stressors may promote a rebound. But the long - term prognosis is uncertain. Like cutthroat populations across the western US, this population faces unknown impacts from climate change^6^. Declining abundance and diverging male and female abundance under changing drought cycles and conditions may have negative long - term consequences. The prediction of increased frequency, severity, and duration of drought, and an increased percentage of rain, may decrease abundance, reduce the effective population size, and skew the sex ratio^5,8,38,55–57,59^. These dynamics may expose this small and isolated population to disproportionately large drift processes (e.g. genetic drift) that could threaten its long-term viability^59^.

## Opportunities for future research

Intertwined, cascading and compounding drought mechanisms in the lake and Mahogany Creek probably caused low survival and recruitment, with evidence at Summit Lake and other trout systems highlighting potential mechanisms that should be investigated. In the lake, evapoconcentration (increased solute concentration and water temperature, and decreased oxygen) may have increased chemical and thermal stress that decreased survival or reproductive readiness, quality, or frequency^30,47,60–63^. In Mahogany Creek, decreased habitat, predation refugia, and post-spawning body condition may have decreased optimal spawning habitat, increased overall spawning mortality, or caused differential spawning mortality between the sexes^7,40,55–57,64–70^. In addition, low fry or juvenile recruitment may have been driven by disturbed stream morphology and ecology that diverted significant streamflow from the main creek channel, which may have physically impeded lakeward migration or decreased instream survival or movement via low streamflows and increased thermal stress^7,24,71,72^.

Utilizing additional or different sampling or modeling techniques should offer additional population dynamics insights by refining estimates and capturing dynamics and mechanisms not addressed in this study. Different sampling techniques are needed to capture juveniles, and sample the heavily vegetated littoral zone and areas further from shore or that are deep. Models that use live/dead or movement/occupancy data could be used to improve abundance and survival estimates^73^. Additionally, a two-sex (to address skewed sex ratios) integrated population model should be considered to perform a population viability analysis. Integrated population models are a relatively new and popular class of population models because they incorporate data from multiple sources to reduce parameter uncertainty^74^. Therefore, future research should focus on the complete life history of the population across time, so that the biotic and abiotic mechanisms driving long - term viability can be understood^6,7,61–63,75^.

## Methods

### Study area and trout population

**Figure 3:**
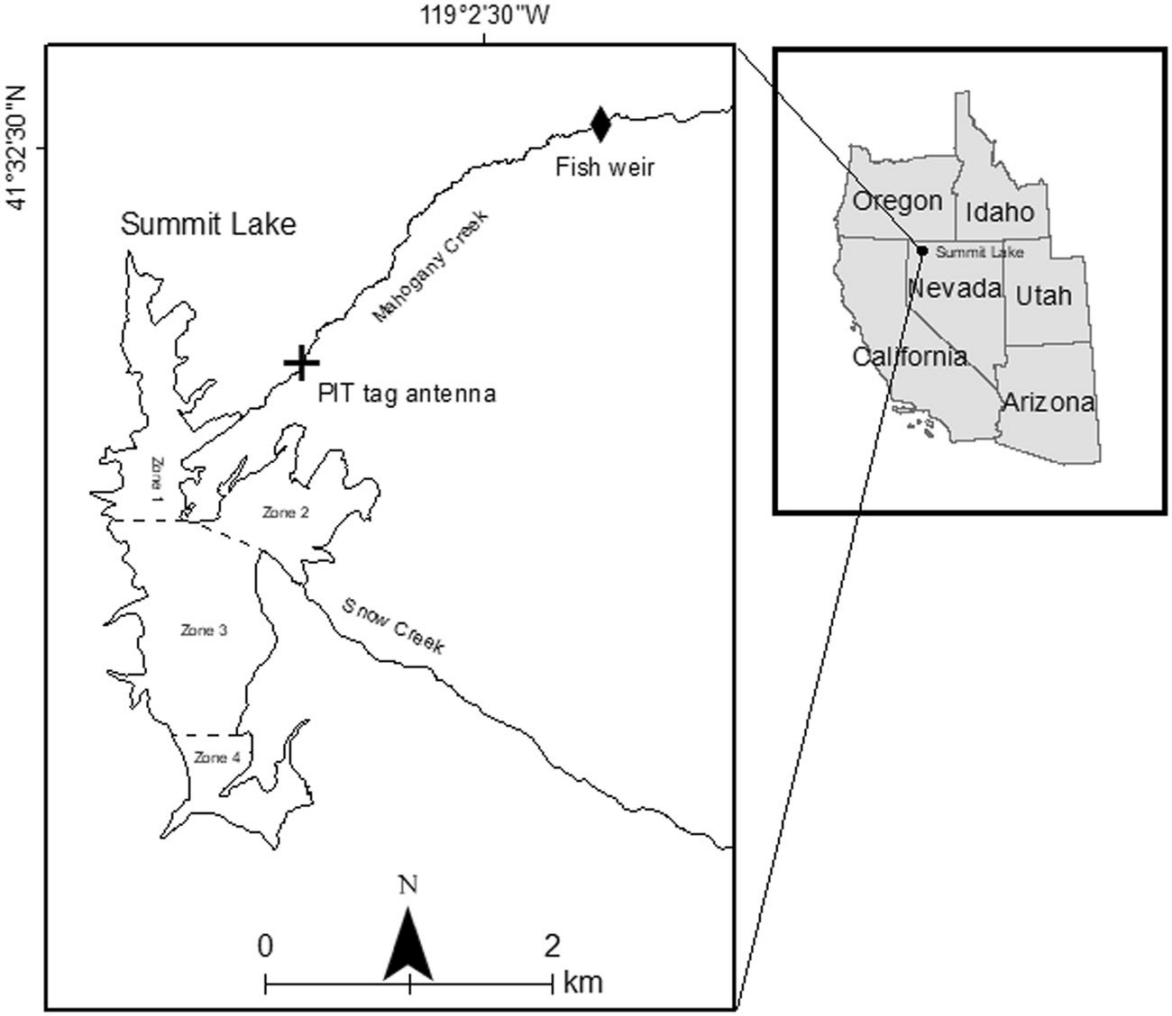
Summit Lake, Nevada, USA, including Mahogany Creek, the sole, perennial spawning tributary. The dashed lines indicate the four sampling zone boundaries, and the cross and diamond represent the PIT tag antenna and fish weir locations, respectively.

Located within the Black Rock Range, Summit Lake (Figs. 1a,b, 3; 41.515 N −119.063 W) is in the northwestern corner of Nevada (Humboldt County), US, at the base of Summit Lake Mountain. Locally, the climate is typical of the Great Basin (warm summers, cold winters, low annual precipitation), sagebrush (*Artemisia tridentata*) is the dominant vegetation, and the geology is tuff, basalt, rhyolite, and pyroclastics^27,76^. The lake and its tributaries are a closed system (i.e., not connected to other surface aquatic systems)^27,28,77^. Because of the lake’s high surface elevation (1780 m) and small surface area (2.8 km^2^), the lake freezes in the winter, stratifies in the summer (surface temperatures >22 °C), and mixes in the spring and fall (i.e., dimictic). The lake is eutrophic, alkaline, and rich in invertebrates and macrophytes^77^. From 1981–1983, surface pH ranged from 8.36–8.54, total dissolved solids (TDS) ranged from 242.4–293.6, specific conductance (μ *mho*/cm @ 25 °C) ranged from 281–355, and total alkalinity (as CaCO_3_) ranged from 128.0–159.2. The lake has a mean depth of 6 m, but the southern half of the lake is generally deeper and contains the maximum depth of approximately 15 m^77^. The lake elevation decreased approximately 4 m (27%) during the recent severe drought in the western US (2012–2016)^54,78^.

Mahogany Creek (Fig. 3), which flows into the northeast shoreline, is the sole perennial spawning tributary and thus serves as the primary source of Lahontan cutthroat recruitment for the lake population^28^. The creek is relatively small, with a mean bankfull width of 2.25 m and mean maximum bankfull depth of 0.42 m^40^. Though spawners utilize most of the creek and its tributaries when conditions permit, most spawning occurs in the lower stretch (approximately 9 km)^78^. Depending on environmental conditions, the spawning run and fry migration can occur from March to June and April to November, respectively^28^. The Summit Lake watershed contains additional creeks that are not perennial, do not have significant spawning runs, or are tributaries to Mahogany Creek.

Public access and fishing have never been allowed on the Reservation. Subsistence fishing is allowed for tribal members but is highly regulated^79^. Invasive salmonids have never been established in the lake or the creeks^28,77^. Egg take and hatchery supplementation of Lahontan cutthroat fingerlings derived from the Summit Lake population were discontinued after 1984^80^. Grazing on the federal and Reservation portions of Mahogany Creek was discontinued in 1974 and 1991, respectively, and in the remainder of the Reservation was minimal from 1990–2004 and henceforth discontinued^28,81^. From 1978 to 2017, the spawning run at the fish weir averaged 1198 ± 583, with a maximum and minimum of 2400 (1999) and 269 (2015), respectively; and from 1997–2017, the spawning run decreased 77% (Supplementary Table S5)^78^.

### Field methods

We conducted this study of Lahontan cutthroat in accordance with the approved procedures in US Fish and Wildlife Permit #TE-17827A-4, and Protocol #00679 of the Institutional Animal Care and Use Committee of the University of Nevada, Reno, US. Lahontan cutthroat are listed as threatened under the US Endangered Species Act. The Summit Lake Paiute Tribe approved the research and protocols and granted access to the Summit Lake Paiute Reservation.

#### Lake mark-recapture effort

We used trap nets for the lake sampling for three primary reasons. Most importantly, as Lahontan cutthroat are threatened, trap nets exert less stress, harm or mortality than other passive methods such as gill nets^82^. Next, trap nets are generally used in shallow water (but can be used up to 15 m) to intercept fish travelling close and parallel to the shoreline, which is ideal because adfluvial Lahontan cutthroat utilize the littoral zone to varying degrees during daily or seasonal cycles^82–85^. In addition, trap nets allowed us to standardize our effort more easily by using the same equipment and length of time repeatedly^82^. Our nets were made of nylon mesh (mesh sizes 13 or 25 mm) with a single leader (1.2 m height, 30.5 m length) in the center of the rectangular throat (1.2 m height × 1.8 m width × 0.6 m length).

We employed a spatially stratified, random sampling plan for this small lake to minimize bias from potential spatial differences in population density (suggested from initial sampling) throughout the lake. Within each primary sampling period (defined below), sampling was performed multiple days a week for consecutive weeks. Each day we deployed between five and ten nets, which were evenly distributed across the four sampling zones (Fig. 3). Primarily, the nets were set perpendicular to shoreline, and most sets were for 20–24 hours. After pulling the nets and processing all captures each day, the nets were moved to a different location within their assigned zone or to another zone to achieve even distribution of effort. For each net set we recorded zone assignment, GPS location, date/time of set and pull, and depth (m).

We anesthetized with CO_2 (g)_ and marked (i.e., tagged) new adult individuals by implanting a Biomark 12 mm full duplex Passive Integrated Transponder (PIT) tag in the pelvic girdle, according to Biomark guidelines^86^. Individuals ≥300 mm were defined as adults based on size data from recent research on the Summit Lake population (Chandra, unpublished data) and literature on Lahontan cutthroat life history^25^. After processing, the fish recuperated in net pens alongside the boat in the lake before release. We recorded the following data from each capture: fork length (mm), mass (kg), new capture or recapture, PIT tag number, and sex (male, female, or unknown).

We determined sex by gamete expression or morphology. During the pre-spawn sampling periods, the anus was gently squeezed from posterior to anterior to express gametes. When gametes were not expressed, and for the other sampling periods, we used visual inspection of head shape/length, overall body shape, and coloration to assign sex, based on the sexual morphology of salmon, brook and bull trout, and Lahontan cutthroat^87–90^. Sex was assigned independently each time an individual was captured. Each capture occasion was reviewed by at minimum two researchers, and unanimous decision was required to assign male or female, otherwise unknown was assigned. At the end of the study, recaptured individuals were coded the sex assigned most frequently. We quantified misidentification by calculating the percentage of instances in which recaptured individuals with known gamete expression had been misidentified. The overall success rate was 79% but with misidentification heavily biased toward females. 35% of female assignments were male, and zero male assignments were female. We used this information to correct any potential influence of misidentification on abundance, survival and spawning estimates (described below)^91^.

To enable precise estimation of abundance and survival rates, our sampling methods followed a standard ‘robust design’ analytical approach; ‘primary periods’ were spaced far enough in time such that births and/or deaths (ignoring migration) were likely to affect population dynamics, and each primary period consisted of two or more ‘secondary periods’ occurring within a short enough time interval to justify a closed population assumption (no births/deaths)^92^. We recognized that the spawning run is a high emigration and mortality event that would violate the assumptions of a closed population model (to estimate abundance, $\hat{N}$) with respect to the lake sampling^43^. Thus, we chose primary periods before and after the spawning run to prevent sampling during the spawning run. Our three annual primary periods were based on meteorological seasons and consisted of ‘pre - spawn’ (March – April, spring), ‘post - spawn’ (June – July, summer), and ‘fall’ (October – November, fall). The daily capture data was consolidated into weekly capture histories. Thus, the secondary periods were consecutive weeks within these primary periods.

We used the CloseTest program, which analyzes a capture history to detect additions or losses, to test that each pre - spawn period was closed^93,94^. The 2015 pre - spawn data indicated losses but no additions, which is in line with the brief overlap of sampling with the beginning of the run. The spawning run had a delayed start likely due to the smallest snowpack (measured in SWE) and lowest streamflow of the study (and drought) period (Supplementary Table S4)^36,54^. Most of the run occurred in late April and May (due to two late precipitation events)^69^. By reviewing the detection record of the PIT antenna, we estimated that seven percent of PIT tagged spawners had migrated into Mahogany Creek during the lake pre - spawn sampling. Thus, we believe the closed population assumption is still reasonable despite a slight potential for positive bias in resulting abundance estimate for the first primary period^95^. In addition, we adjusted (described below) the abundance and confidence interval estimates to account for the bias. The CloseTest results for pre - spawn 2016 and 2017 indicated no additions nor losses. Therefore, no adjustments were made to the estimates for those periods.

The mark-recapture effort was conducted from March 2015 to November 2017 (approximately two years and nine months) for a total of eight primary and 35 secondary (weeks) periods. The number of sampling weeks per primary period ranged from two (pre-spawn 2016 and 2017) to 8 (spring 2015) (mean = 4 ± 2) (Table 1, Supplementary Table S6). We deployed 597 net sets, ranging from 31 (pre-spawn 2016) to 121 (fall 2015) per primary period (mean = 75 ± 38) (Supplementary Datasets S3, S4).

#### Spawning run

A permanent fish weir facility, located approximately 3.5 km upstream from the mouth, captured all spawning trout migrating further upstream. Depending on environmental conditions and the duration of the run, the SLPT generally performed daily checks of the weir from March to June. The following data was collected from each capture: sex (male, female, or unknown), mass (kg), fork length (mm), and PIT number (if present). After processing, the fish were released upstream to continue their migration. Since 1978, the SLPT has enumerated the spawning run at this location^78^.

Because the fish weir is not located at the mouth of the creek, the fish weir counts do not represent the total spawners that participate in the run. Thus, the annual spawner estimates in Mahogany Creek were derived from the annual counts of PIT - tagged spawners detected by a PIT antenna and spawners captured at the fish weir. To generate the counts, we assumed adfluvial Lahontan cutthroat populations have an annual spawning run with a subset of adults, are obligate tributary spawners, and infrequently enter or reside in tributaries outside of the spawning run^25,40^. We classified all detected or captured individuals during the run as a spawner, although actual spawning could not be confirmed. PIT - tagged spawners were detected by a single, stationary PIT antenna (Biomark pass-through antenna with a Biomark IS1001 transceiver) located approximately 750 m upstream from the mouth. The PIT antenna system is automated and records detections 24 hours/day and 365 days/year. The approximate stream width and depth at the location are 74 and 41 cm, and the approximate antenna width and height are 71 and 71 cm, respectively. The detection rate of the antenna was approximately 97%, based on the proportion of PIT - tagged spawners captured at the fish weir that were detected by the PIT antenna^40^.

### Analyses

#### Abundance and survival

We used Program Mark version 9.0 to estimate abundance ($\hat{N}$), seasonal survival (*S*_*s*_), and lambda (λ) for each primary period^96^. We selected the Pradel ‘robust design survival and lambda’ model because we did not need to estimate the additional parameters (e.g., temporary emigration rates) in the standard robust design model^97^. The ‘Huggins’ *p* and *c’* data type (*p* = capture, *c* = recapture) was chosen so that individual covariates (sex, fork length) could be used to model survival, lambda, and capture/recapture^97–99^. In addition, unequal time periods between the primary sampling periods were accounted for in the model setup. The model uses a Horvitz - Thompson estimator to estimate abundance for each primary period^97^. Summit Lake is closed system, and because we avoided sampling during the temporary emigration of the spawning run, our survival estimates represent true survival (*S*) rather than apparent survival^43^.

We processed models with combinations of constant, seasonal, or annual survival, lambda, or capture/recapture rates, and linear, quadratic, or interactive relationships of sex or fork length. We excluded models with non - identifiable parameters. Akaike information criterion (AIC_c_, small sample sizes) model selection was applied to identify the top models^100,101^. We estimated annual survival (*S*_*A*_) as the product of the primary period survival estimates, and used the delta method to estimate standard error for *S*_*A*_^97^.

To examine the sensitivity of our results to sex misidentification, we randomly removed 35% (estimated from our sex misidentification analysis) of the original female capture histories, added them to the original male capture histories (assignment was not changed for individuals of unknown sex), and performed AIC_c_ model selection separately on these adjusted female and male capture histories^91^. We stopped this process after ten iterations because the sex-specific survival and abundance estimates did not change substantially across iterations. We randomly selected one each of the ten alternative male and female capture history databases and combined them with the unknown sex capture history database to estimate total, female and male abundance and survival. Further, sex (male, female, or unknown) was assigned to every individual in this analysis and did not change in the individual’s capture history.

We applied a post-hoc bias correction to our abundance estimate and confidence interval bounds for the first primary period to correct for the estimated 7% of individuals that migrated out of the study population to spawn during this primary period (see above). To do this, we first computed true capture probability (*p*_true_) as *p*_true_ = 1.07 * *p*_1_, where *p*_1_ represents the mean estimated probability of capturing an individual ≥ 1x during the first primary period. We then computed the ‘true’, or bias-corrected, abundance estimate for this primary period by multiplying the abundance estimate N_1_ (estimated abundance for the first primary period) and the corresponding lower and upper confidence bound estimates by the ratio *p*_1_/*p*_true_ [N_true_ = N_1_ ∗ (*p*_1_/*p*_true_)].

#### Spawning run metrics

We accounted for sex misidentification by subtracting 35% of the female counts and adding them to the male counts before performing the below calculations. We estimated the annual count of total, female, and male spawners by dividing the number of tagged spawners detected by the antenna by the proportion of tagged vs. total spawners captured at the fish weir, and then dividing by 0.97 (the detection probability of the PIT antenna)^40^. In addition, total female, male and unknown spawners were estimated by multiplying the total tagged spawners detected by the antenna by the proportion female, male and unknown spawners detected by the antenna. Last, the annual totals were divided by their respective ‘pre-spawn’ primary period abundance estimates to derive the proportion of the adult population that spawned each year.

## Supplementary information

Supplementary Dataset S1.

Supplementary Dataset S2.

Supplementary Dataset S3.

Supplementary Dataset S4.

Supplementary Table S1.

Supplementary Table S2.

Supplementary Table S3.

Supplementary Table S4.

Supplementary Table S5.

Supplementary Table S6.

## Data Availability

All data generated or analyzed during this study are included in this published article [and its supplementary information files].
