# Supplementary material for: Population dynamics of threatened Lahontan cutthroat trout in Summit Lake, Nevada
Source: Sci Rep. 2020 Jun 8;10:9184. doi: 10.1038/s41598-020-65992-0 (PMC7280232; doi:10.1038/s41598-020-65992-0)
Supplement: Supplementary file 5 — Supplementary Table S1. [file 41598_2020_65992_MOESM5_ESM.docx]

Article title: Population dynamics of threatened Lahontan cutthroat trout in Summit Lake, Nevada

Journal name: Scientific Reports

Authors: James B. Simmons, Teresa Campbell, Christopher L. Jerde, Sudeep Chandra, William Cowan, Zeb Hogan, Jessica Saenz, Kevin Shoemaker

Affiliation and e-mail address of the corresponding author: University of Nevada Reno, [jamessimmons@nevada.unr.edu](mailto:jamessimmons@nevada.unr.edu)

**Supplementary Table S1.** Abundance ($\hat{N}$*_t_*), survival (*S_s_*), lambda (λ), and capture (*p_t_*)/recapture (*c_t_*) rate estimates from the top model (Table 2) of adult adfluvial Lahontan cutthroat (male, female, unknown sex, ≥ 300 mm, n = 1082) captured during the lake mark-recapture effort at Summit Lake, Nevada, USA, 2015 – 2017.

| **Parameter** | **Estimate** | **Standard Error** | **95% CI** | |
| --- | --- | --- | --- | --- |
|  |  |  | **Lower** | **Upper** |
| $\hat{N}$_1_* | 1036.46 | 192.92 | 735.76 | 1507.41 |
| $\hat{N}$_2_ | 883.49 | 141.66 | 658.93 | 1223.99 |
| $\hat{N}$_3_ | 850.90 | 115.87 | 661.93 | 1122.17 |
| $\hat{N}$_4_ | 830.37 | 172.40 | 559.49 | 1248.14 |
| $\hat{N}$_5_ | 843.02 | 140.18 | 615.88 | 1173.17 |
| $\hat{N}$_6_ | 933.43 | 149.97 | 691.79 | 1288.49 |
| $\hat{N}$_7_ | 996.91 | 197.45 | 685.64 | 1473.91 |
| $\hat{N}$_8_ | 1085.80 | 216.81 | 747.13 | 1614.00 |
| S_s_ (.) | 0.72 | 0.03 | 0.66 | 0.77 |
| **λ** (.) | 0.99 | 0.03 | 0.13 | 0.99 |
| p_1_* | 0.03 | 0.006 | 0.02 | 0.04 |
| p_2_ | 0.05 | 0.008 | 0.03 | 0.07 |
| p_3_ | 0.14 | 0.02 | 0.11 | 0.19 |
| p_4_ | 0.02 | 0.003 | 0.01 | 0.03 |
| p_5_ | 0.04 | 0.005 | 0.03 | 0.05 |
| p_6_ | 0.10 | 0.02 | 0.07 | 0.13 |
| p_7_ | 0.03 | 0.005 | 0.02 | 0.04 |
| p_8_ | 0.03 | 0.005 | 0.02 | 0.04 |
| c_1_ | 0.03 | 0.007 | 0.02 | 0.05 |
| c_2_ | 0.03 | 0.006 | 0.02 | 0.04 |
| c_3_ | 0.03 | 0.02 | 0.01 | 0.08 |
| c_4_ | 0.06 | 0.02 | 0.03 | 0.12 |
| c_5_ | 0.004 | 0.004 | 0.0006 | 0.03 |
| c_6_ | 0.04 | 0.02 | 0.02 | 0.11 |
| c_7_ | 0.03 | 0.01 | 0.01 | 0.08 |
| c_8_ | 0.006 | 0.004 | 0.002 | 0.024 |

The numbers in the parameter names (ex. $\hat{N}$_1_) indicate the eight primary sampling periods in chronological order and that the parameter is constant within the primary sampling period, (.) indicates the parameter is constant across all primary sampling periods, and CI=confidence interval. *Estimate adjusted to correct for positive bias.
