# Supplementary material for: Population dynamics of threatened Lahontan cutthroat trout in Summit Lake, Nevada
Source: Sci Rep. 2020 Jun 8;10:9184. doi: 10.1038/s41598-020-65992-0 (PMC7280232; doi:10.1038/s41598-020-65992-0)
Supplement: Supplementary file 6 — Supplementary Table S2. [file 41598_2020_65992_MOESM6_ESM.docx]

Article title: Population dynamics of threatened Lahontan cutthroat trout in Summit Lake, Nevada

Journal name: Scientific Reports

Authors: James B. Simmons, Teresa Campbell, Christopher L. Jerde, Sudeep Chandra, William Cowan, Zeb Hogan, Jessica Saenz, Kevin Shoemaker

Affiliation and e-mail address of the corresponding author: University of Nevada Reno, [jamessimmons@nevada.unr.edu](mailto:jamessimmons@nevada.unr.edu)

**Supplementary Table S2.** Adult female abundance ($\hat{N}$*_t_*) estimates derived from the top model (Table 2) of adult adfluvial Lahontan cutthroat (male, female, unknown sex, ≥ 300 mm, n = 1082) captured during the lake mark-recapture effort at Summit Lake, Nevada, USA, 2015 – 2017.

| **Parameter** | **Estimate** | **Standard Error** | **95% CI** | |
| --- | --- | --- | --- | --- |
|  |  |  | **Lower** | **Upper** |
| $\hat{N}$_1_* | 307.72 | 69.27 | 209.56 | 469.80 |
| $\hat{N}$_2_ | 371.55 | 65.66 | 269.24 | 531.91 |
| $\hat{N}$_3_ | 364.94 | 56.03 | 275.48 | 498.75 |
| $\hat{N}$_4_ | 462.49 | 109.43 | 295.71 | 735.14 |
| $\hat{N}$_5_ | 362.90 | 70.12 | 252.76 | 532.87 |
| $\hat{N}$_6_ | 379.92 | 66.86 | 274.56 | 541.48 |
| $\hat{N}$_7_ | 536.12 | 112.72 | 361.25 | 812.49 |
| $\hat{N}$_8_ | 452.79 | 96.05 | 305.66 | 691.00 |

The numbers in the parameter names (ex. $\hat{N}$_1_) indicate the eight primary sampling periods in chronological order and CI=confidence interval. *Estimate adjusted to correct for positive bias.
