# Supplementary material for: Population dynamics of threatened Lahontan cutthroat trout in Summit Lake, Nevada
Source: Sci Rep. 2020 Jun 8;10:9184. doi: 10.1038/s41598-020-65992-0 (PMC7280232; doi:10.1038/s41598-020-65992-0)
Supplement: Supplementary file 7 — Supplementary Table S3. [file 41598_2020_65992_MOESM7_ESM.docx]

Article title: Population dynamics of threatened Lahontan cutthroat trout in Summit Lake, Nevada

Journal name: Scientific Reports

Authors: James B. Simmons, Teresa Campbell, Christopher L. Jerde, Sudeep Chandra, William Cowan, Zeb Hogan, Jessica Saenz, Kevin Shoemaker

Affiliation and e-mail address of the corresponding author: University of Nevada Reno, [jamessimmons@nevada.unr.edu](mailto:jamessimmons@nevada.unr.edu)

**Supplementary Table S3.** Adult male abundance ($\hat{N}$*_t_*) estimates derived from the top model (Table 2) of adult adfluvial Lahontan cutthroat (male, female, unknown sex, ≥ 300 mm, n=1082) captured during the lake mark-recapture effort at Summit Lake, Nevada, USA, 2015 – 2017.

| **Parameter** | **Estimate** | **Standard Error** | **95% CI** | |
| --- | --- | --- | --- | --- |
|  |  |  | **Lower** | **Upper** |
| $\hat{N}$_1_* | 556.82 | 116.93 | 388.27 | 826.19 |
| $\hat{N}$_2_ | 546.88 | 92.32 | 401.58 | 770.23 |
| $\hat{N}$_3_ | 495.55 | 72.71 | 378.41 | 667.71 |
| $\hat{N}$_4_ | 364.39 | 91.86 | 226.64 | 596.69 |
| $\hat{N}$_5_ | 442.74 | 81.91 | 312.87 | 639.56 |
| $\hat{N}$_6_ | 458.87 | 78.39 | 334.54 | 647.20 |
| $\hat{N}$_7_ | 348.48 | 79.60 | 227.44 | 547.25 |
| $\hat{N}$_8_ | 472.76 | 99.69 | 319.83 | 719.70 |

The numbers in the parameter names (ex. $\hat{N}$_1_) indicate the eight primary sampling periods in chronological order and CI=confidence interval. *Estimate adjusted to correct for positive bias.
