# Supplementary material for: Population dynamics of threatened Lahontan cutthroat trout in Summit Lake, Nevada
Source: Sci Rep. 2020 Jun 8;10:9184. doi: 10.1038/s41598-020-65992-0 (PMC7280232; doi:10.1038/s41598-020-65992-0)
Supplement: Supplementary file 8 — Supplementary Table S4. [file 41598_2020_65992_MOESM8_ESM.docx]

Article title: Population dynamics of threatened Lahontan cutthroat trout in Summit Lake, Nevada

Journal name: Scientific Reports

Authors: James B. Simmons, Teresa Campbell, Christopher L. Jerde, Sudeep Chandra, William Cowan, Zeb Hogan, Jessica Saenz, Kevin Shoemaker

Affiliation and e-mail address of the corresponding author: University of Nevada Reno, [jamessimmons@nevada.unr.edu](mailto:jamessimmons@nevada.unr.edu)

**Supplementary Table S4.** 2012 - 2017 snowpack metrics from Summit Lake Mountain, NV, USA, and the annual number of Lahontan cutthroat spawners captured at the Mahogany Creek fish weir at Summit Lake, Nevada, USA^36, 78^.

| **Year** | **Month** | **Snow depth (cm)** | **SWE (cm)** | **Spawning run count** |
| --- | --- | --- | --- | --- |
| 2012 | Jan | 17.78 | 4.83 | 1107 |
|  | Feb | 35.56 | 9.91 |  |
|  | Mar | 63.5 | 14.22 |  |
|  | Apr | 58.42 | 19.05 |  |
|  | May | 0.0 | 0.0 |  |
|  | Jun | 0.0 | 0.0 |  |
| 2013 | Jan | 66.04 | 15.49 | 876 |
|  | Feb | 96.52 | 23.37 |  |
|  | Mar | 83.82 | 24.64 |  |
|  | Apr | 45.72 | 17.78 |  |
|  | May | 0.0 | 0.0 |  |
|  | Jun | 0.0 | 0.0 |  |
| 2014 | Jan | 15.24 | 3.05 | 357 |
|  | Feb | 10.16 | 3.3 |  |
|  | Mar | 30.48 | 8.13 |  |
|  | Apr | 48.26 | 14.48 |  |
|  | May | 0.0 | 0.0 |  |
|  | Jun | 0.0 | 0.0 |  |
| 2015 | Jan | 45.72 | 10.92 | 269 |
|  | Feb | 38.1 | 11.18 |  |
|  | Mar | 20.32 | 5.33 |  |
|  | Apr | 0.0 | 0.0 |  |
|  | May | 0.0 | 0.0 |  |
|  | Jun | 0.0 | 0.0 |  |
| 2016 | Jan | 63.5 | 18.03 | 463 |
|  | Feb | 83.82 | 25.65 |  |
|  | Mar | 73.66 | 26.92 |  |
|  | Apr | 68.58 | 30.99 |  |
|  | May | 0.0 | 0.0 |  |
|  | Jun | 0.0 | 0.0 |  |
| 2017 | Jan | 55.88 | 14.73 | 438 |
|  | Feb | 93.98 | 28.70 |  |
|  | Mar | 114.3 | 35.56 |  |
|  | Apr | 86.36 | 38.61 |  |
|  | May | 55.88 | 25.4 |  |
|  | Jun | 0.0 | 0.0 |  |

Data collected by the Natural Resources and Conservation Service’s SNOTEL station located on Summit Lake Mountain. Data reported in inches but converted to centimeters (cm). SWE=snow water equivalent: the equivalent amount of liquid water.
