# Supplementary material for: Population dynamics of threatened Lahontan cutthroat trout in Summit Lake, Nevada
Source: Sci Rep. 2020 Jun 8;10:9184. doi: 10.1038/s41598-020-65992-0 (PMC7280232; doi:10.1038/s41598-020-65992-0)
Supplement: Supplementary file 9 — Supplementary Table S5. [file 41598_2020_65992_MOESM9_ESM.docx]

Article title: Population dynamics of threatened Lahontan cutthroat trout in Summit Lake, Nevada

Journal name: Scientific Reports

Authors: James B. Simmons, Teresa Campbell, Christopher L. Jerde, Sudeep Chandra, William Cowan, Zeb Hogan, Jessica Saenz, Kevin Shoemaker

Affiliation and e-mail address of the corresponding author: University of Nevada Reno, jamessimmons@nevada.unr.edu

**Supplementary Table S5.** Annual number of Lahontan cutthroat spawners captured at the Mahogany Creek fish weir at Summit Lake, Nevada, USA, 1978 – 2017^78^.

| **Year** | **Spawning run count** |
| --- | --- |
| 1978 | 1763 |
| 1979 | 2380 |
| 1980 | 2122 |
| 1981 | 1176 |
| 1982 | 756 |
| 1983 | 621 |
| 1984 | 639 |
| 1985 | 638 |
| 1986 | 996 |
| 1987 | 855 |
| 1988 | 936 |
| 1989 | 859 |
| 1990 | 718 |
| 1991 | 472 |
| 1992 | 1290 |
| 1993 | 1255 |
| 1994 | 1648 |
| 1995 | 949 |
| 1996 | 1443 |
| 1997 | 1925 |
| 1998 | 1956 |
| 1999 | 2400 |
| 2000 | 2017 |
| 2001 | 1947 |
| 2002 | 1379 |
| 2003 | 1722 |
| 2004 | 1843 |
| 2005 | * |
| 2006 | * |
| 2007 | 950 |
| 2008 | 1030 |
| 2009 | 1160 |
| 2010 | 1150 |
| 2011 | 1008 |
| 2012 | 1107 |
| 2013 | 876 |
| 2014 | 357 |
| 2015 | 269 |
| 2016 | 463 |
| 2017 | 438 |
| MEAN | 1198 |
| SD | 583 |

*Data not collected or available.

SD=standard deviation.
