# Supplementary material for: Population dynamics of threatened Lahontan cutthroat trout in Summit Lake, Nevada
Source: Sci Rep. 2020 Jun 8;10:9184. doi: 10.1038/s41598-020-65992-0 (PMC7280232; doi:10.1038/s41598-020-65992-0)
Supplement: Supplementary file 10 — Supplementary Table S6. [file 41598_2020_65992_MOESM10_ESM.docx]

Article title: Population dynamics of threatened Lahontan cutthroat trout in Summit Lake, Nevada

Journal name: Scientific Reports

Authors: James B. Simmons, Teresa Campbell, Christopher L. Jerde, Sudeep Chandra, William Cowan, Zeb Hogan, Jessica Saenz, Kevin Shoemaker

Affiliation and e-mail address of the corresponding author: University of Nevada Reno, [jamessimmons@nevada.unr.edu](mailto:jamessimmons@nevada.unr.edu)

**Supplementary Table S6.** The start and end dates for the eight primary sampling periods of the lake mark-recapture effort (pre-spawn 2015 to fall 2017) for adult adfluvial Lahontan cutthroat at Summit Lake, Nevada, USA.

| **Primary Period** | **Year** | **Season** | **Start and End Dates** |
| --- | --- | --- | --- |
| 1 | 2015 | Pre-spawn | March 4 – April 26 |
| 2 | 2015 | Fall | October 5 – November 8 |
| 3 | 2016 | Pre-spawn | April 5 – April 14 |
| 4 | 2016 | Post-spawn | May 31 – June 22 |
| 5 | 2016 | Fall | October 17 – November 6 |
| 6 | 2017 | Pre-spawn | March 29 – April 5 |
| 7 | 2017 | Post-spawn | June 12 – July 2 |
| 8 | 2017 | Fall | October 11 – November 8 |
